# Life stories of patients with personality disorders before and after treatment: Change and stability in agency and communion

**DOI:** 10.3389/fpsyt.2023.1134796

**Published:** 2023-03-16

**Authors:** Silvia M. Pol, Fabian Schug, Farid Chakhssi, Gerben J. Westerhof

**Affiliations:** ^1^GGNet Scelta, Apeldoorn, Netherlands; ^2^Department of Psychology, Health, and Technology, University of Twente, Enschede, Netherlands

**Keywords:** life stories, personality disorders, psychotherapy, agency and communion, narrative identity

## Abstract

**Introduction:**

Studying written life stories of patients with personality disorders (PDs) may enhance knowledge of how they understand themselves, others and the world around them. Comparing the construction of their life stories before psychotherapy to their reconstruction after psychotherapy may provide insight in therapeutic changes in the understandings of their lives.

**Methods:**

As few studies addressed this topic, the current study explored changes in agency (i.e., perceived ability to affect change in life), and communion (i.e,, perceived connectedness to other persons) in written life stories of 34 patients with various PDs, before and after intensive psychotherapy treatment.

**Results:**

Life stories showed a positive increase in agency from pre- to posttreatment, in particular regarding internal agency, societal success, and occupational success. No significant changes were observed for communion as a whole. However, the perceived number and quality of close relationships revealed a significant positive increase.

**Discussion:**

The increased agency in the reconstruction of patients’ life story after psychotherapy suggests that patients improved their perceived ability to affect change in their own lives. This can be seen as an important step in the treatment of PDs towards further recovery.

## Introduction

Individuals with personality disorders (PDs) are a heterogeneous group with complex presentations that are characterized by significant distress and/or functional impairment (1). PDs are substantially prevalent in the general population (6.1 to 9.1%; (2, 3)) and in mental health care settings (40–90%; (4)). They are associated with suicide risk (5), high burden of disease and high economic costs for society (6). PDs are maintained by poor metacognition, the capacity to understand mental states of oneself and others for purposeful problem solving, and maladaptive interpersonal schemas, based on experiences and expectations from earlier relationships (7–9). As a result, life stories of patients with PDs would show low levels of agency and communion. Although the effectiveness of psychotherapy for patients with PDs is well documented (10–13), a better understanding of psychotherapeutic change is considered essential for the further development of treatments for PDs (14–17). The therapeutic alliance is considered to be a decisive component of psychotherapy strongly connected to treatment outcome (18). Also, there is moderate to strong evidence that emotional change (regulation, awareness, and transformation), socio-cognitive change (mentalizing, meta-cognition, and interpersonal patterns), and increase in insight and change in defense mechanisms contribute to healthy change in treatment for PDs (19). Based on meta-theoretical models of therapeutic change, (20) proposed a theoretical framework named ‘Agency *via* Awareness’ in which they identify two theoretical assumptions: (1) that increasing agency is a fundamental aim of psychotherapy, and (2) that therapists enhance patients’ agency by increasing their awareness. Life stories of patients with PDs form a reflection of their awareness of their internal and external reality. Therefore, a narrative approach through examining life stories of patients with PDs before and after psychotherapeutic treatment may provide interesting perspectives on change by providing information that standardized assessments or outcome questionnaires might miss.

In the perspective of narrative psychology, human beings give meaning to their life through the narration about themselves, others and the world around them in life stories (21–26). In their life stories, individuals convey to themselves and others who they are now, how they came to be, and where they think their lives may be going in the future (23, 27). This process involves the formation of a narrative identity that provides individuals with a sense of purpose and meaning to their perceived past, present and anticipated future (23, 28–32). Agency and communion are two fundamental and central themes in narrative identity (28, 31, 33). Agency refers to the degree to which individuals are able to affect change in their own lives or influence others in their environment, often through demonstrations of self-mastery, empowerment, achievement, or status. Communion refers to the degree to which individuals demonstrate or experience interpersonal connection through love, friendship or dialog (27). Research has shown that narrative identities high on agentic and communal content are related to higher levels of well-being (34–36). For example, Mendes et al. (24) noted that more reflective narratives and ones which included innovative moments were associated with better therapy outcomes. (37) concluded that an increase in metacognition was connected with symptom reduction at 6 months follow-up. Lack of agentic content in narrative identities is related to the inability to cope with life’s circumstances (28, 38), and an excess of agentic content to overly self-centeredness (39). Lack of communal content in narrative identities is related to the absence of nourishing relationships or presence of toxic relationships, and an excess of communal content to overly selflessness (39–41).

Identity is one of the key-domains that is disturbed in patients with PDs or PD symptomatology, but studies that have examined their narrative identity are scarce (28, 31, 38, 42–45). For example, (28) analyzed life story interviews of 20 adults with features of borderline PD (BPD; i.e., three or more DSM-IV criteria), and a corresponding sample of 20 adults without BPD (i.e., no DSM-IV BPD criteria). They found that agency and communion were significantly less fulfilled among those with than those without BPD features. Similar findings were reported more recently by Lind et al. (45), who examined a sample of 30 adults with BPD and 30 matched controls. Those with BPD, compared to the controls, described their personal life stories more negatively and with fewer themes of agency and communion fulfillment. Kverme et al. (43) interviewed 12 female patients with BPD about their experiences with treatment and recovery, and found that change processes move beyond symptom reduction, and shared the overarching theme of moving toward a personal sense of connectedness. 38 examined narrative identity of five patients with BPD by analyzing the biopsychosocial interview conducted at intake, and the transcription of the first five sessions of psychotherapy. They found that the prevalence of the narrative themes agency and communion was low. Taken together, these findings suggest that lack of agency and lack of communion is prevalent in the narrative identities of patients with PDs, especially with BPD of BPD features. As far as we know, only one study has examined change in narrative identity in patients with BPD after treatment (44). In this study, life stories were analyzed from 23 patients with BPD compared to life stories from 23 control participants, before and after 12 months of psychotherapy. Using a mixture of semi-structured interviews and questionnaires, participants were asked to describe multiple aspects of their life stories which were coded for complexity, emotional valence, agency, and communion. Before therapy, patients with BPD showed low levels of agency and communion fulfillment compared to the controls. After 12 months of therapy, the life stories of the patients with BPD contained significantly more agency and complexity. There were no differences found in the other life story aspects (i.e., communion and emotional valence). The authors conclude that increased agency in life stories may lessen symptoms that are due to an experienced lack of control (such as self-harm or suicidal behavior), and may help patients toward more adaptive behavior.

In 2020 Lind et al. conducted a systematic review over the last decade on how patients with PD and PD pathology construct their narrative identity. The 14 peer-reviewed, English articles used cross-sectional research designs, seldom with a control group of healthy adults. The studies used predominantly categorical operationalizations of PD and were not necessarily carried out in the context of treatment. They asked for personal life stories or significant, self-defining memories by means of semi-structured interviews. The following prominent characteristics of narrative identity were found: (1) motivational/affective themes: negative valence, low agency, low communion fulfillment, and high contamination, (2) autobiographical reasoning: high amount of negative causal connections, and (3) structural elements: mixed results on memory specificity, no significant differences on complexity, low coherence and less life scripts events.

The studies were largely based on female patients with a main diagnosis of BPD. The DSM5 alternative model of personality disorder brought more attention to the sense of self, and authors conclude that more research into narrative identity in PD is needed to learn more about the nature of disordered personality functioning. The aim of this study is to examine change in life stories of patients with PDs before and after psychotherapy, that is the construction and reconstruction of narrative identity, on the themes agency and communion to enhance knowledge of how patients with PDs understand themselves, others and the world around them. The present study differs from the study of (44) in that the life stories were authentically written by patients in a treatment context and were not obtained using a semi-structured interview. In addition to deductive agency and communion coding, we worked with inductive coding which led to a coding schema in which the narratives themes agency and communion were examined in different levels (fulfillment, lack and excess of the theme) and specific meanings, instead of being treated as a single factor.

A qualitative approach was used for describing how agentic and communal content were present in the written life stories of patients with PDs before and after psychotherapeutic treatment. Whereas most studies assessed agency and communion as single dimensions e.g., (28), the current study also took a more bottom-up approach in studying the qualitative content of agency and communion in order to provide more detailed insights into which strengths and challenges people with PDs experience in agency and communion. Next, a quantitative approach was used to examine the changes in prevalence in these narrative themes before and after treatment. In this explorative study our research questions were: (1) Which levels and specific meanings do agency and communion have in life stories of patients in treatment for PD (28, 31, 38, 45), and (2) Which differences in levels and specific meanings do exist in their life stories at the start and end of treatment (43, 44, 46–49)?

## Method

### Setting

This study was conducted in a psychotherapeutic treatment center for patients with PDs that offers residential or day-hospital multidisciplinary group-treatment based on dialectical behavioral therapy (focusing mostly on acquiring skills and works toward practical and emotional stability; (50, 51), and schema focused therapy (focusing mainly on recognizing and adjusting own thought and behavioral patterns, and calls for greater reflection and development of insight; (9, 52)). Participants stayed in residence during 10–12 months (either DBT or ST) or attended weekly 3 days at the specialized day-hospital setting during 9–11 months (combined DBT and ST). The treatment program existed of group therapy (DBT/ST) supplemented by arts therapy, including creative and music, psychomotor psychotherapy, and rehabilitation counseling. A psychiatrist, two clinical psychologists, a creative arts therapist, a psychomotor therapist and psychiatric nurses ran each site. Each day lasted 6 h, divided in the week over group therapy (DBT/ST; 1.30 h.), group therapy (psychodynamic; 1 h.) arts therapy (1 h.), psychomotor therapy (1 h.), rehabilitation counseling (1.25 h.), and divided over the day milieu therapy (1 h. day-hospital, 2 h. residence), lunch break (1 h.), opening- and closing meetings (0.25 h., each). Participants had to attend at least 32 weeks to be considered as someone who completed the treatment, with a maximum of 48 weeks. In previous research, the treatment has proven to be effective in improving personality functioning, well-being and quality of life (51, 53).

### Participants

The majority of the 34 participants were female (79.4%), with a mean age of 32.6 years (SD = 10.4). In all, 35.5% reported completing higher vocational training or university, 23.5% mid-level vocational training, 26.5% upper secondary vocational education and 14.7% pre-vocational secondary education. Among the sample, 47.1% met the DSM-IV diagnostic criteria for PD not otherwise specified, 38.2% for an avoidant PD, 17.7% for a dependent PD, 5.9% for an obsessive–compulsive PD; and 38.2% had traits of Avoidant PD, 26.5% Borderline PD, 17.6% Dependent PD, 11.8% Obsessive–Compulsive PD and 2.9% Narcissistic PD. Also, 88.2% met the diagnostic criteria for comorbid mood disorders: 64.7% for anxiety disorders, 26.5% for eating disorders, 11.8% for psychosocial problems, 8.8% for substance abuse, 8.8% for somatoform disorders and 5.9% for attention deficit and hyperactivity disorder. The majority of the 34 participants followed treatment in the specialized day-hospital setting (55.9%; combined DBT and ST) whereas 20.6%, respectively, 23.5% followed DBT or ST residential treatment.

### Materials

#### Life stories

Participants wrote their life stories in response to the following open-ended question, asked prior to admission to the psychotherapeutic treatment center: “For admission, we would like you to write your life story and a short motivation for your registration for treatment.” After the end of treatment, another question was asked that consisted of the following lines: “We would like you to write your life story again from your current viewpoint, after treatment.” These questions were broadly formulated in order to allow participants to write about their lives in their own words and better understand how participants construct and reconstruct their life story. The sample consisted of 34 pairs of life stories before treatment with a mean of 2,194 words (SD = 2,227; range 267–9,807) and after treatment with an mean of 1,516 (SD = 1,838; range 410–6,368). On the basis of a qualitative assessment, all life stories contained story elements, like a beginning, middle and end with descriptions of personal life experiences. Even though these were not necessarily coherent or complete (54), they provided sufficient information for the analysis of agency and communion.

### Procedure

The study was approved by the ethical review board of the University of Twente, the Netherlands. Patients were eligible to participate in the current study when they met the Diagnostic and Statistical Manual of Mental Disorders, Fourth Edition (55) criteria for at least one personality disorder, as assessed by a psychiatrist or clinical psychologist, based on a clinical interview or previous existing diagnostic information. Participants were recruited from 121 consecutive admissions between December 2014 and April 2016. The drop-out of 41 patients (33.9%) was not unusual for this patient group (56). 51 (42.1%) patients were asked to write their life story after treatment. The main reasons for not asking were that practitioners thought it would ask too much of patients (e.g., due to a worsening condition) or that they had forgotten to ask. 37 (30,6%) patients were willing to write their life story after the end of treatment. Participants were given as much time as needed to finish their life stories. After providing informed consent, 36 participants remained. *A priori* power analysis for a paired t-test with alpha = 0.05, moderate effect size (Cohen’s d = 0.5), and power = 0.80 (G * Power 3) (57) yielded a sample size of 34. Therefore, from the remaining 36 participants, 34 were selected at random. No data is available for the 85 non-respondents because they did not give consent.

### Analysis

#### Qualitative content analysis

Content analysis (58, 59) was used to systematically describe and quantify written life stories before and after treatment (28, 60). Atlas.ti 8 software was used to support the analysis. Meaning units were assigned to all parts of the interviews in consensus by two researchers (SP and FS): a meaning unit for coding consisted of words, sentences or paragraphs containing related aspects in both content and context (61). A hierarchical coding scheme was developed, using deductive (themes of Agency and Communion) and inductive (qualitative content of the themes) procedures. The resulting scheme had two themes, that were subdivided into seven subthemes and 18 codes (see Table 1). The process of content analysis consisted of four steps. The first step concerned the development of a codebook making use of a deductive analysis (theory driven) based on existing definitions of agency and communion (40, 41). The agency theme relates to intellectual desirability, competence, initiating structure, instrumentality, egoistic bias, dominance, and an independent self-construal. The communion theme relates to social desirability, morality, consideration, expressiveness, moralistic bias, nurturance, and an interdependent self-construct. Because of the relatively new sample, patients with PDs, an inductive analysis (data driven) was conducted which led to a distinction between three subthemes: fulfillment, lack and excess of the narratives themes agency and communion, unlike the coding schemas of Adler and McAdams in which these themes were treated as a single factor.

**Table 1:** Themes, subthemes and codes of deductive and inductive content analysis.

Themes	Subthemes	Codes
Agency	Fulfillment of agency	Internal control Initiating support Achievements
Lack of agency	Lack of internal control Lack of contextual control Trauma Failure
Maladaptive coping	Self-mutilation Suicidal behavior Avoidance Other self-destructive behavior
Excess of agency	
Communion	Fulfillment of communion	Presence of relations Quality of relations Empathy
Lack of communion	Missing relations Lack of relatedness Traumatic lack of relatedness
Excess of communion	

Two authors (SP and FS) thoroughly read a sample of six pairs of narratives, pre- and post-treatment, to become completely familiar with the data. They subsequently assigned one or more subthemes to all meaning units. The second step concerned inductive content analysis. The same six pairs of narratives were analyzed and discussed between three authors (SP, SF, and GW), which resulted in a division of the assigned subthemes into more specific codes. Furthermore, several meaning units, that were not assigned to subthemes in step one, were summarized in a new subtheme for agency, i.e., maladaptive coping, which again was subdivided in relevant codes. The third step was an analysis of another sample of nine pairs of narratives. This analysis confirmed the subthemes and related codes. As no new codes arose, saturation was reached and the codebook could be completed with a consensual formulation of codes, and supporting coding rules. The fourth step consisted of the application of the codebook to all 6,311 meaning units in 34 pairs of narratives. Consensual coding, a standard qualitative methodology (62), was used: two researchers (SP and SF) independently coded all meaning units and discussed their coding accordingly until consensus was reached. Each meaning unit could receive multiple codes, i.e., the coding was orthogonal so a total amount of 6,950 was assigned.

#### Quantitative analyses

The percentage of life-stories in which a (sub)theme or code occurs were calculated, as well as the proportion of meaning units in each life-story that were coded with a (sub)theme or code. In this way we corrected for potential length differences between the life-stories before and after treatment. As most of these proportions were not normally distributed, the Wilcoxon signed-rank test, a nonparametric test equivalent to the dependent sample *t*-test, was conducted along with the *t*-test. As the same significant differences were found, the results of the paired *t*-tests were reported for ease of interpretation. Furthermore, effect sizes were calculated using Cohen’s *d* (63), based on post- minus pretreatment scores divided by the pooled standard deviation. Magnitudes of effect sizes (*d*) of >0.56 were considered large, between 0.33 and 0.55 moderate and below 0.33 small (64). The statistical analyzes were performed using IBM Statistical Package for the Social Sciences, version 22 (IBM, 2013).

## Results

### Qualitative content analysis

#### Agency

Content analysis of the theme agency resulted in the emergence of four subthemes and 11 codes (see Tables 1 and 2). The subtheme ‘Fulfillment of Agency’ covers a positive focus on independence and desired goals and directions of life. There are three different aspects of agentic qualities. The first code ‘Internal Control’ describes a broad range of agentic behaviors, like standing up for oneself, perseverance, being in connection with one’s own desires and behavior, being aware of environmental factors and formulating goals and wishes. The second code ‘Initiating Support’ describes the ability to direct oneself toward one’s goals initiating support from family, friends, teachers, healthcare and medication. Persons describe relief when they succeed in arranging help from their environment; when they get parents to talk to school to put an end to being bullied, get a friend to call parents about a crisis, get a general practitioner to refer the person for psychotherapeutic help and get the psychiatrist to prescribe medication that helps them gain control over emotions. The third code ‘Achievements’ addresses positive social and occupational performances in areas of school and education (obtaining diplomas), employment, and living by oneself.

The subtheme ‘Lack of Agency’ describes an experienced lack of governing ability and autonomy and exists of four different codes that all show a shortage in agentic qualities. The first code ‘Lack of Internal Control’ reviews a broad range of experiences of lack of self-governing ability expressed in described complaints, lack of self-efficacy in practical and psychological matters and a lack of problem solving and regulatory skills. The second code ‘Lack of Contextual Control’ describes the perceived lack of influence on external circumstances experienced as negative and disadvantageous but not in strict terms defined as traumatic. The third code ‘Trauma’ concerns the experience of being overwhelmed by severe and dangerous situations such as being bullied, death of loved ones, and/or psychological and physical and/or sexual abuse. The fourth code ‘Failure’ addresses problems with achieving social and occupational performance in the areas of school and education (obtaining diplomas), and work.

Within the subtheme ‘Maladaptive Coping’, a way of coping with life is described that is harmful or has harmful aspects for the person him/herself. There are four codes. The first code ‘Self-Mutilation’ describes behaviors such as hurting oneself by cutting and burning oneself or swallowing large quantities of pills. The second code ‘Suicidal Behavior’ concerns thoughts and actions aimed at ending one’s own life by making scenarios for suicide, taking an overdose of pills or stopping with eating and drinking. The third code ‘Avoidance’ refers to fleeing from problematic emotions and situations, instead of actively tackling these. The fourth code ‘Other Self-Destructive Behavior’ describes a range of other behaviors such as ongoing substance abuse, restrictive or overeating, promiscuous behavior, suppressing feelings and refusing help.

The subtheme ‘Excess of Agency’ describes an excessive focus on autonomy and desired goals at the expense of cooperation with others. It had no further subdivision in codes and describes putting one’s own needs first, while ignoring, neglecting or sometimes even violating the needs of others.

**Table 2:** Subthemes, codes and examples of quotes.

Agency	
Fulfillment of agency	**Internal control**Situations are described such as the narrator called the police, continued to study, got to know herself, initiated talking about emotions, dared to tell others about being abused or was actively involved in sports.	*Although things [at home] were very bad, I never stopped studying. D12-2* *I got to know myself, learned to recognize and set boundaries, learned to feel, to accept myself, finding myself a nice person and much more. D30-2*
**Initiating support**Persons describe that they succeed in arranging help from their environment: they get parents to talk to school to put an end to being bullied, a friend to call parents about a crisis, a general practitioner to refer the person for psychotherapeutic help and a psychiatrist to prescribe medication that helps them gain control over emotions.	*It was so bad that I called my best friend to come and get me. D17-1* *The outpatient care has taught me a lot during the past one and a half years. D1-1*
**Achievements**Experiences are described that lead to a proud sense of achievements in one’s life, such as completing school or university successfully, having an own business, getting promotion in one’s job, teaching or coaching sports in one’s spare time, and managing to get a home of one’s own.	*Meanwhile, I passed my MAVO [secondary school diploma] at the age of 15. D65-1* *I then ran my own kindergarten. D33-1*
Lack of agency	
**Lack of internal control**Persons describe how they have difficulties starting each day, feeling depressed, not knowing how to care for oneself, escaping from daily life through drugs, not being able to tell about being abused, losing the drive to study and being aware of not directing one’s life.	*I never gave direction to my own life. I have never even thought about what this direction should be like. D44-2* *I would like to study, but my insecurity and perfectionism get in the way. D-1*
**Lack of contextual control**Situations stretch from being confronted with illness and missing school, to having traumatized parents, having to deal with parents’ decisions on moving, or parents living according to the rules of strict religion and having a depressed and/or substance abusing partner.	*My parents, who came from families with problems, struggled when I was very young (0-4 years) with psychological problems. For my father, this manifested itself in repeated burnouts, depression and flight behavior. While for my mother, the stress expressed itself in illness and severe depression. D64-2* *He [her partner] became depressed and consumed more alcohol and drugs. D1-1*
**Trauma**Situations are reported of violence and insecurity at home, at school and in the neighborhood, and sometimes the extension of misery in relationships.	*At home, there was much violence and insecurity. D53-1* *I was really bullied at high school. I don’t know the real reason for this but I think it was because I was different from the others. They would wait on me, trip me up, hide my stuff somewhere, follow me around and call me names. I think this period of my life has largely been the cause of my low self-esteem. D25-1*
**Failure**A sense of failure is described around dropping out of school because of too much stress, being suspended from school, or being fired from work because of substance abuse, and losing not only one’s job but sometimes also one’s place in society.	*I was suspended when I was 15 and was told I did not have to come back, so I drank and I smoked hash. D37-1* *In 2014, I became 100% incapacitated for work. On the one side it meant security, on the other side I had to accept that I no longer counted in society. D55-1*
Maladaptive coping	
**Self-mutilation**Behaviors are described such as hurting oneself by cutting and burning, or swallowing large quantities of pills.	*Then it happened more and more, I was obsessed with the color of my blood and how it flowed over my arm and addicted to the burning sensation. D17-1* *I started when I was 15 with scratching and wrapping strings around me (body parts), drinking cleaner and vomiting when I suffered pain or had eaten too much or was called a fat baby. D24-2*
**Suicidal behavior**Thoughts and actions are described aiming at ending one’s own life by making scenarios for suicide, taking an overdose of pills or stopping with eating and drinking.	*The misery at home, boyfriend and school, everything was a total misery, so I (at 16 years of age) took a large dose of pills, I did not want/could not go on anymore. D65-1* *If so, I will put an end to it [my life]. I’ve been thinking this thought for years and about all the possible scenarios for that. D67-1*
**Avoidance**Situations are described like refusing to see reality, continuing to disconnect, fleeing in work or computer games, lying and keeping things hidden from others, not taking time to rest, not being in contact with one’s own feelings, avoiding living during the day, not directing one’s life but just being guided by circumstances.	*I refused to see reality. D8-2* *I have always kept this [abuse by her partner] hidden from family and friends. D4-2*	Maladaptive coping
**Other self-destructive behavior**Behaviors are described like ongoing substance abuse, restrictive eating, sleeping with strangers, repressing feelings and refusing help. Persons are more or sometimes less aware of the self-destructiveness of these behaviors.	*And despite the misery before, especially with alcohol as a cause, I kept drinking a lot in the evenings as well as whole nights. D52-2* *When people wanted to get closer, I would hurt them as much as possible, with the intention that they would never come back. D64-2*
Excess of agency	
Situations are described of putting one’s own needs first, while ignoring, neglecting or sometimes even violating the needs of others.	*My experience in [name city] taught me to put myself first. I had no respect for teachers or other children. D54-2* *[I made myself] big and tough and had a big mouth. D68-2*	Communion
	Fulfillment of communion	
**Presence of relations**Relationships in the narrator’s life are mentioned ranging from parents, partners, brothers and sisters to a school mentor or football coach, general practitioner or psychotherapist, friends and pets.	*After I was born, the three of us were living with my grandfather and grandmother. D15-1* *I got my first horse when I was 11. I could spend the whole day with him. D42-2*
**Quality of relations**Descriptions are found of meaningful experiences feeling safe and respected, being understood, receiving recognition and protection, being offered support and help, doing nice things and working together. Participants describe feeling acknowledged and because of that more strong and resilient, they find their values again and feel that a warm and trustful environment helps them develop.	*When I told about it, I cried really hard but my father responded very well. D15-1* *My social environment is also happy that I express my feelings now. In this way, they also get to see how I am doing and get a better understanding of everything. I have better and a lot more contact with my immediate environment. That is very nice to experience. D26-2*
**Empathy**Persons describe that sometimes behaviors of others, even when experienced as being destructive, can be understood in the circumstances in which the other person lives. Participants describe how this knowledge is important for a better understanding of one’s own experiences and that the destructive behavior of (important) others has nothing or indeed very little to do with the narrator him/herself.	*Our family life was so meagre because my parents had difficulties understanding themselves and choosing their own (family) happiness. D20-2* *My parents did not know what was happening in my life and thought that I was a difficult kid. D36-2*
Lack of Communion	
**Missing relations**Situations are described in which important relations are absent or lost, like fathers being absent, a brother or sister having died, or a friend having moved away.	*I only know that my father was barely home. I frequently did not see him for a couple of days. D2-2* *My younger brother died. D56-2*
**Lack of relatedness**Contact with others is described as (emotionally) distant, emotions are not spoken about, the person feels alone and different from others. Parents have hardly any time for the family or children and are busy with work and other people. There is a remoteness felt with peers and in important relations that is responsible for a feeling of utter loneliness.	*If he was at home, he was just present and that was all that could be said of him. I do not feel like he has really been a father to me. D30-2* *Like my parents, [the therapist] thought it was a puberty thing. In the meanwhile, self-mutilation continued. D6-2*
**Traumatic lack of relatedness**Situations are described of domestic violence and sexual abuse from parents to children and between parents. Also, the abandonment, sometimes by chosen death, by parents has been described as always affecting the narrator’s further life. Peers can be perpetrators when bullying and threatening at school or work, and use extreme violence in relationships. Narrators describe how these traumatic experiences leave them frightened, damaged and completely broken down.	*The images of my mother struck by my father are still imprinted on my eyelids. Broken bones and blood on the wall. D54-2* *I still find it difficult to say his name. He has completely destroyed the self-esteem that I had. D8-2*
Excess of communion	
Situations are described of being insufficiently in contact with one’s own needs, feeling extremely dependent on the opinion and approval of others and/or putting others first.	*He wanted me to be home when he called. Yet he never said what time he would call. So, practically every day I sat waiting for him in my parents’ bedroom from 4 am till 10-11 in the evening. D51-1* *Unfortunately, he drew me into his environment of bunking school, drugs and theft. D45-1*

#### Communion

Analyzing the content of the theme communion led to the emergence of three subthemes and six codes (see Table 1). The subtheme ‘Fulfillment of Communion’ has a positive focus on others for the benefit of relatedness. It offers a rich pallet of impressions on the quality of the relationships described in the narratives. There are three codes within this subtheme. In the first code ‘Presence of Relations’, existing relationships in the narrator’s life are positively mentioned ranging from parents, partners, brothers and sisters to a school mentor or football coach, general practitioner or psychotherapist, friends and pets. In the second code ‘Quality of Relations’, descriptions are found of meaningful experiences with positive emotional relationships with family and family members, partners, peers, teachers, care workers, and the whole of a specific treatment environment. The third code ‘Empathy’ reveals that the behaviors of others, even when experienced as being destructive, can be understood in the circumstances in which the other person lives.

The subtheme ‘Lack of Communion’ has a negative focus on and lack of relatedness with others. It describes a range of lacking qualities in relationships with other persons. In the first code ‘Missing Relations’, the absence of relationships in the narrator’s life is mentioned, like fathers being absent, a brother or sister having died, or a friend having moved away. The second code ‘Lack of Relatedness’ describes the experience of a lack of emotional engagement with others. Contact with others is described as (emotionally) distant, emotions are not spoken about, the person feels alone and different from others. The third code ‘Traumatic Lack of Relatedness’ reviews the realization of a broad range of traumatic experiences of contact with more or less important persons, from severe bullying by peers, traumatic death of loved ones to psychological, physical and/or sexual abuse within or outside the family or intimate relations.

The subtheme ‘Excess of Communion’ describes a focus on others at the expense of one’s own autonomy. It had no further subdivision in codes and describes being insufficiently in contact with one’s own needs, feeling extremely dependent on the opinion and approval of others and/or putting others first.

### Quantitative analysis

The length of the narratives varied widely between participants and showed a reduction after treatment (pre-treatment life stories *M* = 2,194 words, SD = 2,227; post-treatment life stories *M* = 1,516, SD = 1,838; *t*(33) = 0.08; *p* < 0.05). Table 3 provides the prevalence of themes, subthemes, and codes before and after treatment.

**Table 3:** Percentages of narratives, mean proportion of meaning units and standard deviation (between parentheses), paired *t*-test (*t*), and effect sizes (Cohen’s d) for subthemes and codes comparing pre and posttreatment.

Theme							
Subtheme	Code	Pretreatment% narratives	Posttreatment% narratives	Pretreatment*M* (SD)%	Posttreatment*M* (SD)%	*t*		*d*
Agency
Fulfillment of Agency		100	100	29.82 (8.60)	36.69 (12.00)	−2.85	−0.66**	Internal Control	100	100	20.46 (6.24)	30.19 (12.87)	−4.58	−0.96**
Initiating Support	97.1	100	5.93 (5.04)	4.94 (2.21)	1.19	0.26
Achievements	85.3	67.6	3.63 (2.90)	1.65 (1.85)	4.11	0.78**
Lack of Agency		100	100	34.39 (8.20)	27.82 (8.95)	3.83	0.77**	Lack of Internal Control	100	100	23.58 (9.37)	17.85 (8.90)	2.87	0.63**	Lack of Contextual Control	94.1	85.3	8.35 (4.96)	8.87 (7.26)	−0.49	−0.08	Trauma	44.1	35.3	1.53 (2.89)	0.93 (1.45)	1.18	0.26	Failure	38.2	20.6	0.95 (2.28)	0.31 (0.82)	2.05	0.37*
Maladaptive Coping		82.4	85.3	5.62 (5.55)	5.11 (4.56)	0.59	0.1	Self-Mutilation	47.1	32.4	1.14 (2.17)	0.76 (1.39)	1.75	0.21	Suicidality	38.2	38.2	1.44 (3.31)	0.98 (1.82)	1.05	0.17	Avoidance	64.7	79.4	1.93 (2.37)	2.52 (2.09)	−1.54	−0.27	Other Self-Destructive Behavior	32.4	38.2	1.11 (2.27)	0.86 (1.48)	0.57	−0.19
Excess of Agency		17.6	17.6	0.21 (0.55)	0.22 (0.51)	−0.09	−0.02
Communion
Fulfillment of Communion		100	97.1	23.23 (9.75)	24.13 (8.82)	−0.77	0.1	Presence of Relations	97.1	94.1	13.45 (6.67)	10.86 (5.83)	2.53	−0.41**	Quality of Relations	94.1	97.1	9.50 (7.13)	12.68 (7.66)	−2.16	−0.43*	Empathy	17.6	29.4	0.31 (0.97)	0.72 (1.53)	−1.26	−0.32
Lack of Communion		97.1	91.2	13.74 (6.22)	15.66 (7.80)	−1.24	−0.27	Missing Relations	73.5	82.4	1.94 (1.99)	2.56 (2.17)	−1.69	−0.3	Lack of Relatedness	97.1	91.2	10.35 (5.06)	11.34 (6.46)	−0.82	−0.17	Traumatic Lack of Relatedness	44.1	55.9	1.45 (1.66)	1.78 (1.94)	−0.74	−0.14
Excess of Communion		41.2	44.1	0.95 (1.36)	1.41 (2.16)	−1.22	−0.26

*d* = Cohen’s d; SD = Standard Deviation. *p < 0.05. **p < 0.01.

#### Patterns before treatment

The first research question concerned the levels and specific meanings of agency and communion in life stories of patients in treatment for PD. Before treatment, the theme ‘Agency’ occurred in all narratives percentage of Narratives (%Nar 100%) and in a large part of all meaning units (Mean Proportion of Meaning Units (MPMU) 64.21%). Although all participants experienced ‘Fulfillment of Agency’ (MPMU 29.82%), they all struggled with an about equal sense of ‘Lack of Agency’ (MPMU 34.39%). ‘Maladaptive coping’ was described in almost all narratives (%Nar 82.4%), be it in a small proportion of meaning units (MPMU 5.62%), whereas ‘Excess of Agency’ was found regularly (%Nar 17.6%) but in a very small proportion of meaning units (MPMU 0.21%). The subtheme ‘Fulfillment of Agency’ consisted for a large part of the code ‘Internal Control’ (%Nar 100%; MPMU 20.46%) and to a lesser degree of the codes ‘Initiating Support’ (%Nar 97.3%; MPMU 5.93%) and ‘Achievement’ (%Nar 85.3; MPMU 3.63%). The subtheme ‘Lack of Agency’ showed roughly the same pattern for the codes ‘Lack of Internal Control’(%Nar 100%; MPMU 23.58%), ‘Lack of Contextual Control’ (%Nar 94.1%; MPMU 8.35%), and ‘Failure’ (%Nar 38.2%; MPMU 0.95%). The code ‘Trauma’ (%Nar 44.1%; MPMU 1.53%) as well as the codes of the subtheme ‘Maladaptive Coping’ occurred in a substantial part of the narratives, but only in a small proportion of the meaning units; Self-Mutilation (%Nar 47.1%; MPMU 1.14%), Suicidality (%Nar 38.2%; MPMU 1.44%), Avoidance (%Nar 64.7%; MPMU 1.93%), and Other Self-Destructive Behavior (%Nar 32.4%; MPMU 1.11%). These findings answer the first research question, lack of agency (%Nar 100%; MPMU 34.39%) is prevalent in life stories, and fulfillment of agency (%Nar 100%; MPMU 29.82%), was found to an almost equal amount. Furthermore trauma (%Nar 44.1%; MPMU 1.53%) played a role in many life stories and maladaptive coping (%Nar 82.4%; MPMU 5.62%) in most life stories.

The theme ‘Communion’ (%Nar 100%) also occurred in all narratives before treatment and although less than agency, it was present in a substantial part of all meaning units (MPMU 36.97%). Participants described the ‘Fulfillment of Communion’ somewhat more often (%Nar 100%; MPMU 23.23%) than ‘Lack of Communion’ (%Nar 97.1%; MPMU 13.74%). The subtheme ‘Excess of Communion’ was described in a substantial part of the narratives (%Nar 41.2%), but in a very small proportion of meaning units (MPMU 0.95%). The subtheme ‘Fulfillment of Communion’ consisted for an important part of the codes ‘Presence of Relations’ (%Nar 97.1%; MPMU 13.45%) and ‘Quality of Relations’ (%Nar 94.1%; MPMU 9.50%), whereas ‘Empathy’ did not occur often (%Nar 17.6%; MPMU 0.31%). The subtheme ‘Lack of Communion consisted mostly of the code ‘Missing Relations’ (%Nar 73.5%; MPMU 1.94) and for an essential part of the code ‘Lack of Relatedness’ (%Nar 97.1%; MPMU 10.35%), whereas the code ‘Traumatic Lack of Relatedness’ occurred in a substantial part of the narratives (%Nar 44.1%) but only in a small proportion of the meaning units (MPMU 1.45%). Lack of communion is quite prevalent (%Nar 97.1%; MPMU 13.74%), but communion fulfillment is even more central to the life stories (%Nar 100%; MPMU 23.23%).

#### Changes in pattern

The second research question concerned the differences in levels and specific meanings in the life stories at the start and end of treatment.

A paired *t*-test was conducted to compare subthemes and codes in life stories before and after treatment (see Table 3). ‘Fulfillment of Agency’ (*d* − 0.66, *p* < 0.01) was significantly higher after treatment than before. However, the specific meaning of this subtheme changed as well: ‘Internal Control’ (*d* − 0.96, p < 0.01) showed a significant increase, whereas ‘Initiating Support’ remained unchanged and ‘Achievement’ (0.78, *p* < 0.01) revealed a significant decrease. The subtheme ‘Lack of Agency’ (0.77, *p* < 0.01) was significantly lower after treatment. This was in particular related to the decrease in ‘Lack of Internal Control’ (0.63, *p* < 0.01) and ‘Failure’ (0.37, *p* < 0.05), whereas ‘Lack of Contextual Control’ and ‘Trauma’ remained unchanged. The subtheme ‘Maladaptive Coping’ and its codes as well as the subtheme ‘Excess of Agency’ remained unchanged. These findings answer the second research question, there are higher levels of agency after treatment, whereby the specific meaning of agency has changed toward the experience of more internal control, less lack of internal control and failure, but also less experience of achievement.

The proportions of the subthemes ‘Fulfillment of Communion’, ‘Lack of Communion’, and ‘Excess of Communion’ all stayed the same. The prevalence of codes mostly remained the same although there is a shift in focus from the ‘Presence of Relations’ (−0.41, *p* < 0.01) to the ‘Quality of Relations’(−0.43, *p* < 0.01). These findings answer the second research question; there is not more fulfillment of communion, even though the specific meaning of communion has changed toward a significant positive increase in the perceived number and quality of close relationships.

## Discussion

The aim of this study was to advance the understanding of how patients with PDs construct their own life story before and after psychotherapy. To this end, the content, prevalence and changes in agency and communion in written life stories were explored before and after psychotherapy of 34 patients with PDs. These themes were studied in different qualities, resulting in subthemes of fulfillment, lack and excess of the theme as well as more specific codes, in contrast to other studies that assessed agency and communion as dimensional constructs e.g., (28). In answer to the research question, a lack of agency and communion was prevalent, although fulfillment of agency was equally prevalent and fulfillment of communion was even more prevalent than lack of communion. In answer to the second research question, there was an increase in fulfillment of agency, but no change in communion as a whole, although the perceived number and quality of close relationships revealed a significant positive increase.

The prevalence of lack of agency and communion in the life stories of patients with PDs corresponds with earlier studies on life stories of patients with PDs, although the patients in the current study had a wider variety of PDs than existing studies that focused mainly on borderline PD (28, 31, 38, 42, 45). Before therapy, patients with PDs have a narration of themselves, others, and the world around them that is marked by an impaired orientation on goals, skills and accomplishments, and a lack of safe embedding in social connections. However, it should be noted that fulfillment of agency and communion also played an important role in the stories of patients with PDs, a finding that has not been stressed in previous studies. These findings underline the relevance of giving explicit attention to positive characteristics in treatment to improve well-being and recovery in patients with PD (65).

Furthermore, the content analysis showed that subthemes and codes from the inductive analysis provided richer information about the narratives of persons with PD. In particular, agentic and communal aspects of maladaptive coping strategies and trauma contain meanings of agency and communion that might be specific for persons with PD. The pattern of maladaptive coping strategies, together with traumatic experiences and lack of contextual control, can be interpreted as an attempt to make use of agentic qualities to regulate or avoid overwhelming negative emotions. Also, absence of relations, together with a lack of relatedness and traumatic interpersonal experiences, may contribute to significant impairments in interpersonal functioning. These findings connect to the DSM5 Alternative Model for Personality Disorders in which PDs are defined on a dimensional factor of “moderate or greater impairment in personality (self/interpersonal) functioning” (1), p. 775, provide a rich perspective on these characteristics, and show the value of the inductive approach that was used in the content analysis.

In the reconstruction of their life stories after treatment, patterns of themes revealed positive changes in fulfillment of agency as found by (45). Content-wise, participants were less occupied with success and failure, and were feeling more capable of directing their lives (i.e., improved agency) toward their values. The patterns of themes revealed no change in communion as a whole as earlier found by (44), but the perceived number and quality of close relationships revealed a significant positive increase. The results seem to suggest that improvement in perceived ability on pursuing goals and manifesting skills and accomplishments (agency) and forming and maintaining social connections (communion) are important factors in the recovery of patients with PDs. These results match with a review and previous study on recovery for persons with PDs in which safety, social relationships and autonomy are decisive factors to recovery (19, 43, 49). And the results are in line with the ‘Agency *via* Awareness’ framework by (20) in which increasing awareness is assumed to lead to increasing agency. Also, psychotherapy outcome research utilizing the Core Conflictual Relationship Theme (CCRT), shows that mastering negative relationship patterns is supporting in developing better relationship patterns with oneself and others (7). Interestingly, the life stories written after treatment were shorter in length. Patients seemed less preoccupied with certain aspects of their lives and events seemed more integrated. This would suggest changes in emotional valence, complexity, and coherence that could be addressed in further research (31).

This study has certain limitations. It examined life stories before and after therapy in a specific setting and did not use a control group which would have strengthened the conclusions that can be drawn. Furthermore, no clinical measures on level of personality functioning and severity of symptoms could be obtained for every participant which hampers the generalization of the findings. For future studies, it is recommended to study change in narratives in connection with psychotherapy outcomes and measures of personality, so that change can be understood from different perspectives e.g., (66). In this study the sample did only comprise patients having completed treatment which might be the most resourceful patients. As is well-known in clinical practice for PDs, women were overrepresented: future studies could include an analysis of gender differences. In order to collect authentic life stories, the instruction for participants was unstructured and open. Also, the writing process at two moments in time is subject to context effects. These circumstances resulted in large fluctuations between narratives in length and content, making it more difficult to compare the narratives, and draw conclusions about narrative change. In the present study participants were instructed to generate written life stories, but existing studies indicate that an oral versus written narrative format may influence the results (42, 48). Rasmussen used interviews to allow for more detailed and extensive narratives and to check for possible differences in written language skills between participants, but he noticed that interviews also allowed participants to generate more off-target information about events, possibly resulting in slight differences between the findings of both studies. Therefore, we cannot exclude that participants were hindered by writing skills that influenced the results. The patients in this study received either DBT and/or ST and it would have been interesting to examine whether one of the treatments were more efficient on improving agency than the other. For this, psychotherapy outcomes measures as well as a larger number of patients in each condition is needed. The categories “excess of agency and communion” offer an interesting perspective as these might be more prevalent among certain PD subgroups than others. For example, focusing on others at the expense of one’s own autonomy might be more prevalent in people with dependent PD. The research by (67) suggests that developing a healthier narrative would be associated with: (a) increased reliance on autobiographical memory instead of semantic reasoning (b) richer descriptions of mental states, (c) passing from predictions that some innermost wishes will remain unmet to ideas that they will be eventually met. For future studies it would be very interesting to examine if these processes are related to quantitative and qualitative changes in Agency and Communion. Although linguistic approaches offer a fundamentally different, albeit complimentary, approach to operationalizing identity (31), a consideration for future studies can be to conduct text analysis based on theoretically driven codes to add to qualitative analysis. An example is the Computerized Reflective Functioning (CRF) that measures the stylistic dimension of reflective functioning by calculating the frequency of some linguistic markers (47).

Nevertheless, a strength of this study is its clinical sample of patients with PDs treated in clinical practice. Another strength is the authenticity of the analyzed material; the participants had the freedom to write their life story without a given structure, which led to autonomous and highly personal accounts of their lives. Also, the narratives themes agency and communion were studied in different qualities (fulfillment, lack and excess of the theme) and not treated as a single factor.

This study may have some clinical implications. First, clinicians may explicitly pay attention to patients’ perceived ability of agency and communion, and which experiences may enhance agency and communion that may further their recovery. Second, therapeutic approaches that directly focus on agentic qualities, such as emotion regulation skills and problem solving skills are recommended. Third, directly improving the quality of relations of patients with PDs may be an important element to focus on for treatment, as patients with PDs are known to have difficulties with building trust and stability in relationships (28). Fourth, stronger agentic qualities and supporting relationships may help patients with PDs to overcome the lasting influence of difficult and painful experiences in the past. Finally, the reconstruction of the narrative identity in patients with PDs may provide these individuals with understanding and acceptance of their past, and a sense of purpose and new perspectives regarding their present and their future.

## Data availability statement

The original contributions presented in the study are included in the article, further inquiries can be directed to the corresponding author.

## Ethics statement

The studies involving human participants were reviewed and approved by the ethical review board of the University of Twente, the Netherlands. The patients/participants provided their written informed consent to participate in this study.

## Author contributions

SP, FS, and GW contributed to the conception and design of the study and wrote sections of the manuscript. FS and SP organized the database and performed the statistical analysis. SP wrote the first draft of the manuscript. All authors contributed to manuscript revision, read, and approved the submitted version.

## Conflict of interest

The authors declare that the research was conducted in the absence of any commercial or financial relationships that could be construed as a potential conflict of interest.

## Publisher’s note

All claims expressed in this article are solely those of the authors and do not necessarily represent those of their affiliated organizations, or those of the publisher, the editors and the reviewers. Any product that may be evaluated in this article, or claim that may be made by its manufacturer, is not guaranteed or endorsed by the publisher.
